# The Accidents Which May Happen to Ovarian Cystic Tumours

**Published:** 1908-02-01

**Authors:** 


### Gynæcology.

### THE ACCIDENTS WHICH MAY HAPPEN TO OVARIAN CYSTIC TUMOURS.

Apart from the fact that ovarian cysts and adenomata are fatal tumours if left alone, and, apart from the dangers of malignant change, ovarian cysts may menace life by reason of the accidents to which they are liable. The common accidents to which these growths are exposed are torsion of the pedicle, rupture of the cyst, and infection of the cyst. These lesions are all of most serious import in a woman who is known to have an ovarian tumour, but when they occur to a patient who is not known to be so subject their danger is greatly increased. The reason for this is that in all these accidents the patient is acutely ill, and the difficulty of diagnosis is rendered very great on account of the pathological effects of the accident.

Torsion of an ovarian pedicle is sometimes definitely associated with pregnancy or labour, and under these conditions may be mechanically explained. It, however, occurs sufficiently often in women who are not pregnant to be regarded as no rarity, and in such cases the causation is certainly mysterious. It has been ascribed to sudden movements of the diaphragm or abdominal muscles, and possibly the varying degrees of intestinal distension may assist in its causation. Often, however, no real cause can be found for the accident. The pathological effect of torsion of the pedicle is interference with the blood supply of the tumour. The tightness of the twist will account for the more or less severe onset of symptoms, and the direct closure of the veins by twisting will lead to the well known congestion of the tumour, and hæmorrhage into its substance. This hæmorrhage has been known to be so severe as to cause rupture of the cyst and fatal syncope, though such a result is very uncommon.

The twisted tumour, being almost in a condition of blood stasis, is very easily infected by organisms from the intestines, and peritonitis on its surface with formation of adhesions results. Should the patient survive the toxæmia necessarily thus set up, it is probable that partial gangrene of the tumour, or at least suppuration, will occur. This result, however, is not often seen except in very chronic cases, when the symptoms are at first slight. As a rule they are so marked, and the patients are so acutely ill, that advice is sought early, and operative interference takes place. The symptoms occasionally start from the very moment of the accident, sudden pain and collapse marking the time at which the twist occurs. But as a rule the twist does not set in with dramatic suddenness: it is more gradual, and the symptoms may not be marked until peritonitis occurs or hæmorrhage into the tumour causes some anæmia.

There is nothing distinctive in the symptoms, as they are those of local peritonitis, and are common to all the acute abdominal conditions which are accompanied by peritoneal infection. If, however, there is sudden pain, rigidity of the abdominal muscles, high temperature, vomiting, and some degree of anæmia, in a person who is known to have an ovarian tumour, torsion of the pedicle is highly probable. If examination reveals for the first time the presence of a tumour arising out of the pelvis, although the characteristics of an ovarian cyst may be obscured by the acute symptoms of its torsion the diagnosis should at least be suspected. It occasionally happens in more chronic cases that large quantities of peritoneal fluid are thrown out, completely distending the abdomen, and making it impossible to feel any tumour. The diagnosis is then very difficult, but as such a case usually calls for surgical relief, the condition will be found on opening the abdomen.

When a twisted cyst is diagnosed, the only treatment is removal of the tumour, and the sooner the better. It makes no difference if the patient is pregnant, or has been just delivered: the presence of a pregnant uterus scarcely complicates the operation at all, and a considerable number of cases have been operated upon without even causing abortion. The results of operation in these cases are very good, in spite of the infection, and no drainage of the peritoneum is required unless pus has formed outside the tumour, an exceedingly rare complication.

Rupture of an ovarian cyst is a much rarer condition than torsion of the pedicle, but has a somewhat similar causation. It is sometimes caused by pregnancy and labour, but may also be the result of direct violence from some sudden movement or strain. The diagnosis will always be difficult, and sometimes impossible, unless it can be shown that a tumour, which was known to exist, has suddenly disappeared. If a unilocular cyst with watery contents bursts it may do no harm, the fluid being absorbed and excreted by the kidneys, the cyst refilling later. A multilocular cyst rarely does more than burst one of its cavities, and the pseudo-mucinous contents being of an irritating nature produce signs of peritoneal irritation. A papillomatous cyst, on the other hand, may only burst in the sense that the solid masses may grow through the cyst wall and disseminate the new growth all over the peritoneum. The symptoms which may then be expected will be sudden pain on rupture, perhaps an increased urinary flow some hours later, and more or less peritoneal irritation. Such a history will usually call for an exploratory laparotomy, for the patient may be alarmingly ill at first, and the diagnosis may be complicated by suspicions of ruptured extra-uterine gestation, ruptured gastric ulcer, acute appendicitis, or some form of intestinal strangulation. Infection of an ovarian cyst is a rare condition comparatively speaking, apart from axial rotation, and surface infection resulting in adhesions. It may occur as a result of intestinal adhesion with migration of bacteria from the bowel to the cyst, and it has been described as occurring in connection with zymotic diseases such as typhoid. All adhesions on ovarian cysts are the result of infection, but in this connection we are more particularly concerned with infection of the growth itself resulting in suppuration. The association of high fever, wasting, and leucocytosis with a pelvic cystic tumour will always suggest suppuration and call for radical treatment.

